# Leprosy in the elderly population of an endemic state in the Brazilian Northeast (2001–2017): epidemiological scenario^☆^^☆☆^

**DOI:** 10.1016/j.abd.2019.01.011

**Published:** 2019-12-18

**Authors:** Carlos Dornels Freire de Souza, Tânia Rita Moreno de Oliveira Fernandes, Thais Silva Matos, Clódis Maria Tavares

**Affiliations:** a Fundação Oswaldo Cruz, Rio de Janeiro, RJ, Brazil; b Medical Program of Universidade Federal de Alagoas, Arapiraca, AL, Brazil; c Universidade Federal de São Paulo, São Paulo, SP, Brazil; d Medical Program of Universidade Federal do Vale do São Francisco, Petrolina, PE, Brazil; e Program of Biological Sciences and Health of Universidade Federal do Vale do São Francisco, Petrolina, PE, Brazil; f Dr. Altino Lemos Santiago Leprosy Reference Center, Juazeiro, BA, Brazil; g Universidade de São Paulo, São Paulo, SP, Brazil; h School of Nursery and Pharmacy, Universidade Federal de Alagoas, Maceió, AL, Brazil

**Keywords:** Aging, Leprosy, *Mycobacterium leprae*

## Abstract

This ecological study aims to analyze both the tendency and the characteristics of leprosy in the elderly population in the state of Bahia, 2001–2017. The tendency was analyzed through joinpoint regression. Epidemiological variables were also included in the study. The average detection rate was 38.73/100,000, with prevalence of men (45.19/100,000). A downward trend occurred in both genders, from 2004, with a greater magnitude in women (annual percent change [APC] = −3.4%). Men presented higher proportions of the multibacillary forms and physical disabilities. The epidemiological scenario indicates the need of implementation of actions that stimulate early diagnosis and treatment of the elderly population.

Brazil contributed with 92.3% of new leprosy cases in the Americas region. The three countries (Brazil, Indian, and Indonesia) with the highest burdens accounted for 80.2% of the new case load globally in 2017.^1^ In 2017, Brazil recorded 26,875 new cases of the disease, 2225 of which (8.28%) occurred in Bahia's residents.^2^

Aging is characterized by important physical, functional, biological and psychosocial transformations that increase the risk of developing some diseases.^3^ Therefore, the aging process of the Brazilian population, a consequence of the demographic transition, justifies the need of understanding how leprosy has been affecting this age range. Moreover, the gender-stratified analyses, according to the World Health Organization (WHO) recommendation, have a special relevance, because they document the nuances of the disease process in each population subgroup.^1^

Thus, the objective was to analyze epidemiological tendency and characteristics of leprosy in the elderly population, from 2001–2017, in the state of Bahia, Brazil.

An ecological study was conducted. The data related to the new cases were obtained from the National System of Notification Diseases (Sistema Nacional de Agravos de Notificação [SINAN]), and the population data from the Brazilian Institute of Geography and Statistics (Instituto Brasileiro de Geografia e Estatística [IBGE]), accessed in March 2018.

The first step consisted of the analysis of the tendency of detection rate of new cases, stratified according to gender. The joinpoint regression model was used.^4^ The tendency was classified as increasing, decreasing, or stationary. In addition, the annual percent change (APC) and the average annual percent change (AAPC) were calculated with 95% confidence intervals (95% CI). Authorization by the Research Ethics Committee was not required, because the data used are secondary.

The second step consisted of the epidemiological analysis of the following variables: gender, age, race/color, schooling, clinical form, operational classification, detection mode, and degree of physical incapacity at the time of diagnosis.

There were 8843 new cases of leprosy recorded in the elderly Bahia population. The average detection rate was 38.73/100,000 inhabitants, with greater detection in men (45.19/100,000 inhabitants) when compared to the women (33.54/100,000 inhabitants). Considering that the number of leprosy cases in Brazil has been contested by researchers,^5^ the actual epidemiological scenario of leprosy in the elderly may be even more worrying.

The joinpoint model showed two distinct temporal trends. The first indicated growth (2001–2004), and the second demonstrated reduction (2004–2017), with a greater magnitude of reduction in women than men (APC = −3.4 and −2.2%, respectively), corroborating other studies.^3^, ^6^, ^7^ At the same time, when considering the tendency of the total period (2001–2017), the observed trend was stationary (Fig. 1).

**Figure 1:**
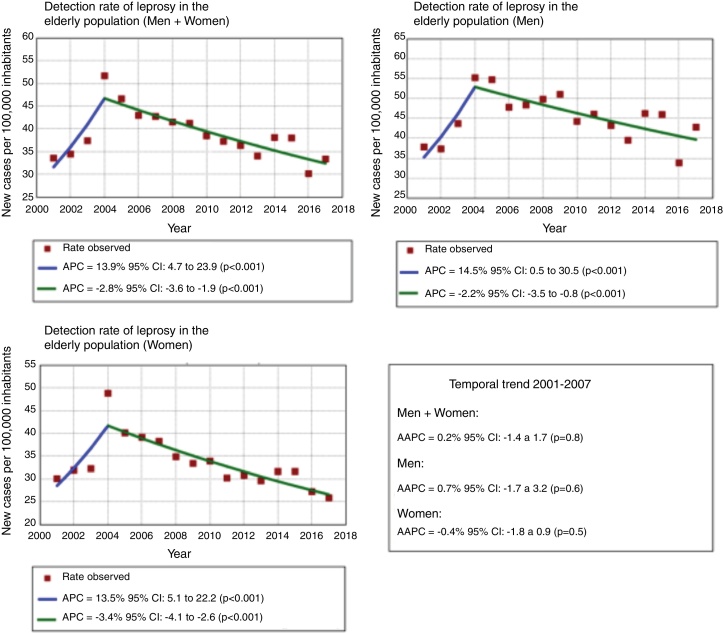
Trend of the detection rates of new cases of leprosy in the elderly population, stratified by gender. Bahia, Brazil, 2001–2017. APC, annual percent change; AAPC, average annual percent change; CI, confidence interval.

With regard to the epidemiological characteristics, a slight predominance of cases in the male population was detected (52.13%, *n* = 4610), age between 60 and 69 years old (57.86%, *n* = 5117), brown-skinned (50.42%, *n* = 4459), and low level of schooling, with 27.77% (*n* = 2456) being illiterate. 52.26% (*n* = 4621) were diagnosed by means of referral (Table 1). Similar results were evidenced in other investigations.^3^, ^8^, ^9^

**Table 1:** Sociodemographic and clinical characterization of leprosy cases diagnosed in the elderly population. Bahia, Brazil, 2001–2017.

Variable	Male *n* (%) 4610 (52.13%)	Female *n* (%) 4233 (47.87%)	Total *n* (%) 8843 (100%)
*Age range*			
60–69	2691 (58.37%)	2426 (57.31%)	5117 (57.86%)
70–79	1423 (30.87%)	1263 (29.84%)	2686 (30.38%)
80 or more	496 (10.76%)	544 (12.85%)	1040 (11.76%)

*Ethnicity*			
White	965 (20.93%)	897 (21.19%)	1862 (21.06%)
Black	754 (16.36%)	646 (15.26%)	1400 (15.83%)
Yellow	25 (0.54%)	29 (0.68%)	54 (0.61%)
Mixed-race	2356 (51.11%)	2103 (49.68%)	4459 (50.42%)
Indigenous	20 (0.43%)	13 (0.31%)	33 (0.37%)
Ignored/left blank	490 (10.63%)	545 (12.88%)	1035 (11.70%)

*Schooling*			
Illiterate	1220 (26.46%)	1236 (29.20%)	2456 (27.77%)
Elementary school	2073 (44.97%)	1777 (41.98%)	3850 (43.54%)
Middle school	258 (5.60%)	236 (5.57%)	494 (5.59%)
High school	81 (1.76%)	63 (1.49%)	144 (1.63%)
Ignored/left blank	978 (21.21%)	921 (21.76%)	1899 (21.43%)

*Clinical type*			
Indeterminate	404 (8.76%)	545 (12.88%)	949 (10.73%)
Tuberculoid	676 (14.67%)	1085 (25.63%)	1761 (19.91%)
Dimorphous	1536 (33.32%)	1290 (30.47%)	2826 (31.96%)
Virchow's	1.047 (22.71%)	502 (11.86%)	1549 (17.52%)
Ignored/left blank	947 (20.54%)	811 (19.16%)	1758 (19.88%)

*Functional classification*			
Paucibacillary	1087 (23.58%)	1859 (43.92%)	2946 (33.31%)
Multibacillary	3509 (76.12%)	2352 (55.56%)	5861(66.28%)
Ignored/left blank	14 (0.30%)	22 (0.52%)	36 (0.41%)

*Method of detection*			
Referral	2431 (52.73%)	2190 (51.74%)	4621 (52.26%)
Walk-in	1652 (35.84%)	1466 (34.63%)	3118 (35.26%)
Collection-based exam	127 (2.75%)	121 (2.86%)	248 (2.80%)
Contact-based exam	235 (5.10%)	321 (7.58%)	556 (6.29%)
Others	117 (2.54%)	102 (2.41%)	219 (2.48%)
Ignored/left blank	48 (1.04%)	33 (0.78%)	81 (0.92%)

*Grade of physical incapacitation*			
Grade 0	2299 (49.87%)	2491 (58.85%)	4790 (54.17%)
Grade I	1050 (22.78%)	881 (20.81%)	1931 (21.84%)
Grade II	507 (11.00%)	228 (5.39%)	735 (8.31%)
Ignored/left blank	754 (16.35%)	633 (14.95%)	1387 (15.68%)

In the gender-stratified analyses, men presented higher proportions of the multibacillary forms when compared to the female population (3509/76.12% and 2352/55.56%, respectively). The lepromatous clinical form corresponded to 22.71% (*n* = 1047) of the registered cases in the male population and only 11.86% (*n* = 502) of the registered cases in the female gender (Table 1). These data suggest that diagnosis occurs later in men,^10^ possibly due to two factors: less access to health services and the effects of negligence regarding symptoms of the disease.^3^, ^8^, ^9^

The presence of physical disabilities was another element that was highlighted in the analyses. Of the total number of new cases, 30.15% (*n* = 2666) already had some incapacity at the time of diagnosis and, in 8.31% (*n* = 735), these disabilities were permanent. The proportion of men with permanent physical incapacity was 2.04 times higher than that of women (11.00%, *n* = 507 and 5.39%, *n* = 228, respectively) (Table 1). This scenario highlights what has been mentioned previously, indicating the severe consequences of the disease in the elderly population,^3^, ^9^ especially in the male population, and in the maintenance of the chain of transmission in the community.^5^

Although the results presented are already capable of justifying the authors’ concern about leprosy in the elderly population, the high proportions of unfilled and/or ignored fields in the evaluation of the degree of physical disability at the time of diagnosis reflect not only on the operational problems in disease vigilance,^3^, ^6^, ^9^ but also indicate that the number of disabled may be even higher than that presented.

Leprosy in the elderly must be viewed with concern, since this population is stricken by more multibacillary forms of the disease and has an increased risk of developing physical disabilities. In addition, the precarious access to health services – which makes these patients invisible – keeps the disease's chain of transmission active, making it persistent in the community.

## Financial support

None declared.

## Authors’ contributions

Carlos Dornels Freire de Souza: Statistical analysis; approval of the final version of the manuscript; conception and planning of the study; composition of the manuscript; collection, analysis, and interpretation of data; participation in the design of the study; intellectual participation in the propaedeutic and/or therapeutic conduct in the studied cases; critical review of the literature; critical review of the manuscript.

Tânia Rita Moreno de Oliveira Fernandes: Approval of the final version of the manuscript; conception and planning of the study; composition of the manuscript; participation in the design of the study; intellectual participation in the propaedeutic and/or therapeutic conduct in the studied cases; critical review of the literature; critical review of the manuscript.

Thais Silva Matos: Statistical analysis; approval of the final version of the manuscript; conception and planning of the study; composition of the manuscript; collection, analysis, and interpretation of the data; participation in the design of the study; intellectual participation in the propaedeutic and/or therapeutic conduct in the studied cases; critical review of the literature; critical review of the manuscript.

Clódis Maria Tavares: Approval of the final version of the manuscript; conception and planning of the study; composition of the manuscript; participation in the design of the study; intellectual participation in the propaedeutic and/or therapeutic conduct in the studied cases; critical review of the literature; critical review of the manuscript.

## Conflicts of interest

None declared.
